# A Macrophysiological Analysis of Energetic Constraints on Geographic Range Size in Mammals

**DOI:** 10.1371/journal.pone.0072731

**Published:** 2013-09-13

**Authors:** Salvatore J. Agosta, Joseph Bernardo, Gerardo Ceballos, Michael A. Steele

**Affiliations:** 1 Center for Environmental Studies and Department of Biology, Virginia Commonwealth University, Richmond, Virginia, United States of America; 2 Department of Biology, Texas A&M University, College Station, Texas, United States of America; 3 Southern Appalachian Biodiversity Institute, Roan Mountain, Tennessee, United States of America; 4 Departamento de Ecología de la Biodiversidad, Instituto de Ecología, Universidad Nacional Autonoma de Mexico, México D.F., Mexico; 5 Department of Biology, Wilkes University, Wilkes-Barre, Pennsylvania, United States of America; The University of Wollongong, Australia

## Abstract

Physiological processes are essential for understanding the distribution and abundance of organisms, and recently, with widespread attention to climate change, physiology has been ushered back to the forefront of ecological thinking. We present a macrophysiological analysis of the energetics of geographic range size using combined data on body size, basal metabolic rate (BMR), phylogeny and range properties for 574 species of mammals. We propose three mechanisms by which interspecific variation in BMR should relate positively to geographic range size: (i) Thermal Plasticity Hypothesis, (ii) Activity Levels/Dispersal Hypothesis, and (iii) Energy Constraint Hypothesis. Although each mechanism predicts a positive correlation between BMR and range size, they can be further distinguished based on the shape of the relationship they predict. We found evidence for the predicted positive relationship in two dimensions of energetics: (i) the absolute, mass-dependent dimension (BMR) and (ii) the relative, mass-independent dimension (MIBMR). The shapes of both relationships were similar and most consistent with that expected from the Energy Constraint Hypothesis, which was proposed previously to explain the classic macroecological relationship between range size and body size in mammals and birds. The fact that this pattern holds in the MIBMR dimension indicates that species with supra-allometric metabolic rates require among the largest ranges, above and beyond the increasing energy demands that accrue as an allometric consequence of large body size. The relationship is most evident at high latitudes north of the Tropics, where large ranges and elevated MIBMR are most common. Our results suggest that species that are most vulnerable to extinction from range size reductions are both large-bodied and have elevated MIBMR, but also, that smaller species with elevated MIBMR are at heightened risk. We also provide insights into the global latitudinal trends in range size and MIBMR and more general issues of phylogenetic and geographic scale.

## Introduction

“Who can explain why one species ranges widely and is very numerous, and why another allied species has a narrow range and is rare?” - Darwin 1859:6 [1]

A fundamental task of Ecology is to understand the distributions of species. In particular, the puzzle of why even closely related species vary so greatly in the size, limits, position, and shape of their distributions has been an enduring concern of ecologists [2-7]. For example, geographic range size (hereafter, range size) varies by 12 orders of magnitude among extant terrestrial mammals [8].

Attempts to understand determinants of range properties have been pursued at many geographical and phylogenetic scales. At one extreme are analyses of one to a few species using correlations between climatic variables (e.g., temperature isoclines) and range edges, or mechanistic analyses using experimental approaches such as transplants beyond a range margin or evaluation of abiotic tolerances [9-12]. Although case studies may clarify mechanisms, they typically lack generality because so few species are studied.

At the opposite extreme are macro-scale approaches, our focus in this study, generally involving large datasets of hundreds to thousands of species within large taxonomic groups such as mammals, birds, reptiles or palms, over continental scales [4,13-18]. Both approaches have implicated a wide array of intrinsic factors, such as physiological tolerances, life history and trophic position; and extrinsic factors, such as the presence and number of competitors, predators and parasites [2,7,19,20] as determinants of the size, position and limits of the range. Yet after more than a century of study, and an expansive catalog of case studies and correlations of range properties with many variables [19], recent reviews highlight our limited understanding of the underlying causal mechanisms that determine this variation at all scales [7,19,21,22].

Macroecological studies have advanced our understanding of species distributions by quantitatively and statistically describing range properties for many kinds of organisms, and how these properties vary with species traits, geography, habitat and taxonomy. However, the success of macroecological approaches in concretely explaining range properties has been limited, for several reasons. First, variables included in macroecological analyses such as body size or trophic position typically explain small amounts of total variation (e.g., Table 1). Second, macroecological studies still largely ignore phylogenetic relatedness as a confounding factor in statistical analysis of pattern, which is now well recognized as an important issue when using a comparative approach [1,23,24].

**Table 1:** Amount of variation in log_10_ geographic range size explained as a linear function of log_10_ mass in various groups of mammals and New World birds.

Group	No. species	% variation explained (R^2^)	Source
Amazonian primates	39	19	[137]
New World carnivores	70	9	[62]
Neotropical forest mammals	100	10	[138]
African large mammals^a^	242	4	[139]
Carnivora	210	4.3	[8]^b^
Primates	259	4.8	[8]^b^
Rodentia	1,287	n.s.^c^	[8]^b^
Mammalia	3,268	n.s.	[8]^b^
New World birds	2,908	1	[117]

Geographic range size was measured as squared area, except where noted. Only studies that measured the complete extent of geographic ranges, rather than partial or regional ranges, were included. Note the overall negative correlation between the number of species included in the study and amount of variation explained.

^a^ latitudinal extent was used as the measure of range size

^b^ calculated from all available data in the PanTHERIA database [8]

^c^ n.s. = not significant (p > 0.05)

Finally, most macroecological studies infer mechanisms tangentially, either because they are derived from correlations to proxy variables such as latitude, or from coarse scale correlations with habitat variables. For example, many recent studies have used correlational habitat modeling and latitude to infer that ‘physiological tolerance’ explains variation in range size [25,26]. But organisms generally experience climate on a much finer scale than is captured by the ‘average climate’ at a given latitude and temperature moments (mean or daily and seasonal variance) can be complexly and nonlinearly related to latitude [27,28]. In addition, many other factors that likely contribute to range size besides temperature covary with latitude, including land area, productivity, species diversity, and resource turnover rates. Moreover, ‘physiological tolerance’ is a complex multivariate species trait with many distinct cellular mechanisms that evolve more or less independently (e.g., heat tolerance, cold tolerance, tolerance breadth, etc.). Any one or some combination of these underlying mechanisms of tolerance could drive patterns. In the end, such habitat correlational studies can only provide statistical support for correlations between range properties and coarse-scale abiotic factors, an understanding that was well-articulated more than a century ago [29-31].

The emergent field of Macrophysiology aims to address this lack of mechanism by incorporating physiological species traits (PSTs) that are theoretically related to both environmental tolerance/performance and energetics into analyses of range properties [32-36]. PSTs that are hypothesized to relate to range properties include heat and cold tolerance limits, tolerance breadth, performance capacity, performance breadth, and acclimation capacity [32,37-42]. Thus macrophysiology integrates several longstanding research approaches to species distributions including the mechanistic perspective of physiological ecology, the comparative insights amassed by comparative physiology, and the large scale of analysis inherent in macroecology [36].

In this paper, we take a macrophysiological approach using data on basal metabolic rate (BMR) to understand mammalian distributions, a group that has been of seminal import in macroecological studies. Mammals are also well-studied physiologically, and several explicit hypotheses exist about how and why variation in BMR should relate to mammalian distributions. Additionally, we take a phylogenetic approach to examine whether accounting for relatedness alters conclusions. Finally, we explore relationships between physiology and range size at different taxonomic and geographic scales to assess how the scale of comparison influences the hypothesized functional relationships.

### Theoretical basis of, and alternative hypotheses for, a functional relationship between PSTs and range size

The thesis that physiology interacts with climate to determine range size has deep historicalroots (e.g., [29,43,44]; reviewed by [36]), but a focus on biotic interactions marginalized physiological thinking from mainstream ecology for decades [36]. Macrophysiology [36] provides a framework for evaluating functional hypotheses about how PSTs relate to range properties.

To date, macrophysiological studies are limited and focus on the role of physiological niche breadth, primarily tolerances to temperature. The Climatic Variability Hypothesis [43] predicts that species that experience wider variation in climate (e.g., temperate zone terrestrial species) should have evolved broader physiological tolerances [28,36,43-46] and therefore realize larger ranges (reviewed by [45]) than related species occupying less variable climates. However, few quantitative tests of these predictions actually exist [7,22,36,45]. The few extant studies of ectotherms find the expected positive relationship between interspecific variation in thermal tolerance and latitudinal position of the range [37,47,48] and between thermal tolerance and range size [21,27,38,49,50]. Other aspects of physiological tolerance, such as resistance to water logging and drought stress in grasses [51], also positively correlate with range size.

Little attention has been given to how PSTs other than tolerance, such as species differences in basal metabolic rates (BMR), might influence range size or position [32] despite the well-developed theoretical connection of metabolism and overall energy budgets [52-58]. This is particularly surprising for mammals, which have been a favorite subject of macroecology and comparative physiology, and for which there is statistically significant residual interspecific variation in BMR not explained by body size allometry [59,60]. Moreover, the energetics of endothermy and its relationship to both energy supply and demand have been widely studied (see below), and have been hypothesized previously to explain the classic macroecological relationship between body size and range size in mammals and birds [14,18]. This relationship is positive and conforms roughly to a triangular ‘constraint space’ [14,16,61,62], where small species can achieve small or large ranges, but as species get larger, they are increasingly restricted to larger ranges. The most widely accepted explanation for this pattern is that, because larger organisms require more energy, they are constrained to have lower population densities, larger home ranges and thus larger geographic range size to maintain minimum viable population sizes to avoid extinction [14,61,63-65]. Below we refer to this as the Energy Constraint Hypothesis. Despite continued interest in the body size-range size relationship over the last two decades, little new insight into this macroecological pattern has emerged (but see [65]), in the sense that fuller statistical explanations of the variance in range size have not been realized (Table 1). Moreover, although effects of body size on metabolic rate and energetics are well-described for mammals, the role of PSTs and BMR in particular as predictor variables of range size has not yet been assessed directly, despite the fact that the original explanation for the body size-range size relationship was stated in explicit energetic terms [18].

BMR is a measure of the rate of energy use necessary for sustaining basic physiological functions [66-68]. In endotherms, our focus here, it is defined as the minimal rate of metabolism during rest in a postabsorptive (non-digestive) state measured within the thermoneutral zone [69]. Because BMR is strongly positively correlated with body size in animals [70,71], any macroecological relationship between BMR and range size should be similar to the previously well-known relationship between body size and range size discussed above. Of more interest here is that, across the range of mammal body sizes and taxonomic diversity in the present data set (see Methods), there is ~6% unexplained residual variation in BMR with respect to mass (hereafter, mass-independent BMR, MIBMR). MIBMR correlates negatively with environmental temperature and positively with latitude in mammals [39,67,72-75].

Latitude is often used as a proxy for thermal environments because average ambient temperatures (*T*_env_) generally decrease and temperature variation (∆*T*_env_) generally increases from the equator to the poles (Figure 3 in [76]; see also [27]). Selection for increased heat production to maintain body temperature in the face of low *T*_env_ and high ∆*T*_env_ is thought to be the primary driver of the latitudinal gradient in MIBMR.

We hypothesize three related, non-mutually exclusive causal links between species differences in BMR and range size:

1 *Thermal* *Plasticity* *Hypothesis*: High BMR increases thermal tolerance [66,77], which increases potential range size. Given sufficient available energy, high BMR species can better maintain homeothermy at high latitudes and elevations and are better equipped to cross physiological barriers, such as mountain chains [43,45,78], with comparatively low *T*_env_ and high ∆*T*_env_. As a result of their increased aerobic scope (i.e., ability to increase metabolism above resting rate), high BMR species are also better able to harvest enough energy in the short-term to meet energy requirements in the long-term, such as hoarding food for winter. Thus, high BMR species are better equipped to inhabit more variable thermal and resource environments, such as those typically found at higher latitudes and elevations [77]. Nonetheless, high BMR species are not necessarily restricted to living at high latitudes or elevations because genetic, phenotypic and seasonal variation in metabolism, body size and heat dissipation mechanisms (e.g., fur length or density) can allow populations at low latitude and low elevation to persist.
2 *Activity* *Levels /Dispersal* *Hypothesis*: High BMR increases activity levels and dispersal potential of individuals and, ultimately, increases potential range size. Intraspecific and interspecific studies of diverse animal taxa generally indicate a positive link between BMR and activity levels [73,79-85], metabolic rates and dispersal potential [32,86,87], and dispersal potential and range size [32,51,88-90]. Moreover, the evolution of endothermy and associated high BMR is most often explained by the aerobic capacity model [77,91,92], which states that high BMR evolves as a correlated response to selection for increased aerobic scope to power higher maximal rates of metabolism or higher rates of sustained metabolic activity. At the cellular level, high BMR permits higher rates of ATP synthesis and more rapid use of ATP by muscles and visceral organs to support higher rates of foraging, growth, reproduction and thermogenesis [77]. Presumably, the increased aerobic scope associated with increased BMR allows individuals to move over larger distances, forage and seek mates more widely, and disperse and colonize at higher rates. Low BMR is advantageous because it requires less energy, which may permit occupation of low-energy habitats; however, the cost is that physiological rates and behaviors governing growth, activity and reproduction are reduced, and thus rates of dispersal and colonization are potentially low.
3 *Energy* *Constraint* *Hypothesis* [14,18,61,63-65]: Because higher BMR requires higher, sustained levels of energy throughput, habitat energy availability should constrain the evolution, interspecific frequency, and geographic distribution of high BMR. Concomitantly, once high BMR evolves, these same demands may constrain individuals to forage farther and space themselves more widely to obtain sufficient energy, resulting in low population densities, larger home ranges, and ultimately larger species distributions to maintain minimum viable population sizes to avoid extinction. As discussed above, this mechanism has been proposed previously to explain body size-range size relationships [14,18,61,63-65].

**Figure 1:**
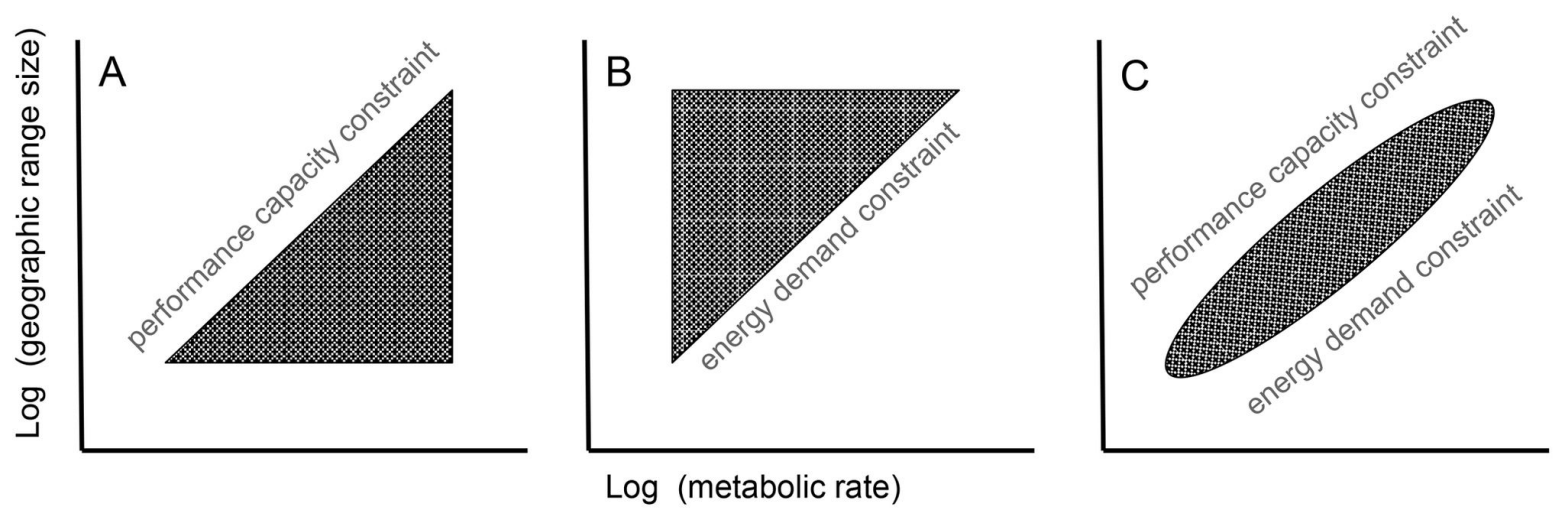
Hypothetical forms of a positive macroecological relationship between basal metabolic rate (BMR) and geographic range size. (a) Expected relationship if low BMR (low performance capacity) is a constraint on achieving large range size (upper bound with positive slope) and high BMR is an advantage for achieving large range size, but not a constraint on persisting with small range size (constant lower bound). This pattern would be consistent with the Thermal Plasticity Hypothesis and Activity Levels/Dispersal Hypothesis. (b) Expected relationship if high BMR (high energy demand) is a constraint on persisting with large range size (lower bound with positive slope), but not a requirement for achieving large range size (constant upper bound). This pattern would be consistent with the Energy Constraint Hypothesis, and is the same pattern described previously for the relationship between body size and range size. (c) Expected relationship if constraints on range size operate at both ends of the BMR spectrum. At low BMR, species are constrained against achieving large ranges as in (a). At high BMR, species are constrained to have large ranges as in (b). See text for details.

These non-mutually-exclusive hypotheses can be partially contrasted based on the form of the macroecological pattern they predict (Figure 1). The Thermal Plasticity Hypothesis and Activity Levels/Dispersal Hypothesis can both be viewed as constraints arising from performance capacity and predict that low BMR is a constraint on achieving large range size, but that high BMR species can have large or small ranges. These hypotheses predict a positive relationship described by a triangular trait-space lacking species with low BMR and large range size (Figure 1a). In contrast, the Energy Constraint Hypothesis is a constraint arising from energy demand and predicts that high BMR is a constraint on persisting with small range size, but not a requirement for achieving large range size, so that low BMR species can have large or small ranges. This hypothesis predicts a triangular trait-space lacking species with high BMR and small range size (Figure 1b). If these mechanisms operate together, then we should observe an absence of both species with low BMR and large ranges and species with high BMR and small ranges. In this case, the relationship should be well-described by a line (Figure 1c).

This paper has three major aims. First, we test for the predicted positive relationship between BMR and range size and attempt to resolve the three hypotheses outlined above. To do this, we analyzed a relatively large sample of terrestrial mammals (45% of which are rodents), taking account of phylogeny and covariation with latitude and body size. Second, we evaluate the global latitudinal trends in mammal range size and MIBMR and relate these to climate and geographic differences in the MIBMR-range size relationship. Third, we provide a general commentary on the value of a macrophysiological approach for understanding species distributions and the broad conservation implications of our study for predicting vulnerability to range size reductions driven by large-scale environmental change (e.g., habitat loss, climate change).

## Methods

### Data sources

Range size in this study represents an estimate of the area contained within the outer-most limits of a species’ known distribution [7]. Range size, along with latitudinal and longitudinal extents of occurrence, were obtained from the PanTHERIA database [8], with modifications by G. Ceballos (see refs [93,94] for details) for 4,668 extant, non-marine mammal species. Body size (mass) and BMR data that met standards for reliability per Sieg et al. [60] were available for 695 species (Dryad Digital Repository: http://datadryad.org/handle/10255/dryad.712). The final data set used in this study included 574 species for which there were combined data on range size, mass and BMR, and which were included in the most complete mammalian phylogeny available (which includes 4,510 extant species and is 47% resolved compared to a fully bifurcating tree [95,96]). The phylogeny and all species names follow Wilson & Reeder [97]. We examined two nested data sets, all mammals (N = 574) and rodents (N = 259).

### Statistical analysis

All variables were log_10_ transformed. To derive MIBMR, we regressed BMR against mass to obtain the residuals. This was done separately for the analysis involving all mammals and that involving only rodents. We used non-phylogenetic ordinary least squares regression (OLS) and phylogenetic generalized least squares regression (PGLS) to test for a relationship between MIBMR and range size. Although MIBMR was our variable of central interest, we conducted similar analyses of mass and BMR versus range size. We first used OLS to examine latitudinal trends in range size and MIBMR. We assigned species along the latitudinal gradient using the latitudinal midpoint of a species geographic range [7]. Given that range size was significantly related to latitude, we used the residuals of this relationship to obtain latitude-independent range size for our most conservative test of the MIBMR-range size relationship.

Because shared evolutionary history among species can cause interspecific trait similarity, it may inflate statistical power in comparative analyses, possibly leading to erroneous conclusions [23,24,98,99]. Nevertheless, current methods for accounting for phylogenetic autocorrelation in comparative studies (e.g., PGLS) are not a panacea for resolving these issues because (1) most phylogenies are incomplete and include unresolved relationships (‘soft’ polytomies), and (2) specific models of trait evolution, such as Brownian motion, must be assumed to perform the analyses, yet are not empirically supported [100]. For comparison and because neither method is ideal, we used both OLS and PGLS regression in this study.

To perform PGLS, we used Pagel’s correlation structure (corPagel) in the APE package [101] in the program R 2.11.1 [102]. Pagel’s version of PGLS (PGLSλ) assumes a Brownian motion model of evolution to estimate the amount of phylogenetic signal in the trait data (λ) using maximum-likelihood and then uses this value to estimate the phylogenetically-corrected regression between traits [98,103,104]. The parameter λ usually varies between 0 and 1, but in some circumstances can be > 1. When λ = 0, there is no phylogenetic correlation in the trait data and those species can be treated as independent observations (trait variation is independent of phylogeny). In this case, PGLSλ is equivalent to OLS. When λ = 1, this indicates that traits have evolved along the phylogeny in a manner congruent with Brownian motion, such that trait variation scales in direct proportion to shared evolutionary history. In this case, PGLSλ is comparable to independent contrasts [23]. Intermediate values of λ can be interpreted as the strength of the phylogenetic signal in the trait data from 0 to 1. Because the species-level relationships of mammals are only ~50% resolved, and therefore phylogenetic comparative methods may allow only weak tests under these circumstances, we also examined relationships among families – both across mammals and within rodents – to provide an additional perspective on the role that phylogenetic relatedness may play in these data.

We first regressed range size against mass, BMR and MIBMR without respect to latitude to compare the nature and strength of the relationships. For a more conservative test of the MIBMR-range size relationship, we accounted for covariance between range size and latitude by regressing residuals of the range size-latitude relationship (latitude-independent range size) against MIBMR. We examined this relationship globally and for three regions: North (> 23.7° latitude), Tropics (23.7° to -23.7° latitude), South (< -23.7° latitude). Species were assigned to regions based on the latitudinal midpoint of their range. Third, we used OLS and PGLSλ multiple regression to test for an effect of MIBMR on range size with mass and latitude as additional predictors and to examine the predictive power of multivariate models. Because the previous analyses indicated significant differences, we conducted separate multiple regressions for each region. Lastly, we used OLS simple regression to examine the relationship between MIBMR and latitude-independent range size at the among-families level using family mean trait values.

In all analyses, we used the Akaike Information Criterion with the second-order correction for finite sample sizes (AICc) to choose between linear and curvilinear models when fitting OLS regressions and to evaluate whether OLS or PGLSλ provided a better fit to the data [105]. All statistical analyses were performed in R 2.11.1 or JMP 9.0.0 [106] and were considered significant at p < 0.05. p-values between 0.05 and 0.10 are referred to as ‘marginally significant’.

## Results

Our data set comprised 99 mammal families, 39% of which were represented by a single species. The representative number of species for the remaining families ranged from 2-76. In the full mammal data set, range size (as area, km^2^) is highly correlated with both latitudinal (r = 0.95, p < 0.0001) and longitudinal (r = 0.94, p < 0.0001) extent of geographic ranges. Thus, range size in squared area, and latitudinal and longitudinal extent provide very similar information. We present only the results involving range area.

Histograms of range size are shown in Figure S1. Range size in mammals varied from less than 1 km^2^ for the murid rodent *Melomys* *rubicola* to 63 million km^2^ for the red fox ( *Vulpes* *vulpes* ). By contrast the distribution of range sizes of the 574 species for which we had appropriate metabolic rate data ranged from 2,237 km^2^ in the chipmunk *Tamias* *palmeri* to 63 million km^2^ in the red fox. Thus, our data set varied over 5 orders of magnitude compared to 12 orders of magnitude in PanTHERIA and was biased against species with small ranges. Both distributions were strongly right-skewed for untransformed data but less strongly left-skewed for log_10_ transformed data. Similar results were found when the rodent data were analyzed separately (Figure S1).

The allometries of BMR and mass and the residuals are shown in Figure S2. For all mammals, mass explained 94.5% of the variation in BMR. Even on a log-log plot, the allometry was best described by the quadratic function: log_10_BMR = 0.642 + 0.673 · log_10_ Mass + 0.031 · (log_10_ Mass - 2.269)^2^ (F_2, 571_ = 4897.08, p < 0.0001). Although the improvement in R^2^ was negligible (< 1%) compared to the linear function, the quadratic term was significant, so we used it to derive the residuals for our measure of MIBMR (results were qualitatively the same using the residuals estimated from the linear function). Residuals were normally distributed and MIBMR was not correlated with mass; species with both higher and lower than expected BMR for their body size occurred across the range of masses (Figure S2). For rodents only, mass explained 88.4% of the variation in BMR: log_10_BMR = 0.694 + 0.671 · log_10_ Mass (F_1, 257_ = 1962.75, p < 0.0001). Again, residuals were normally distributed and not correlated with mass.

Although MIBMR and mass were orthogonal, MIBMR and BMR were correlated for all mammals (r = 0.23, n = 574, p < 0.0001) and for rodents only (r = 0.32, n = 259, p < 0.0001).

### Latitudinal trends

**Figure 2:**
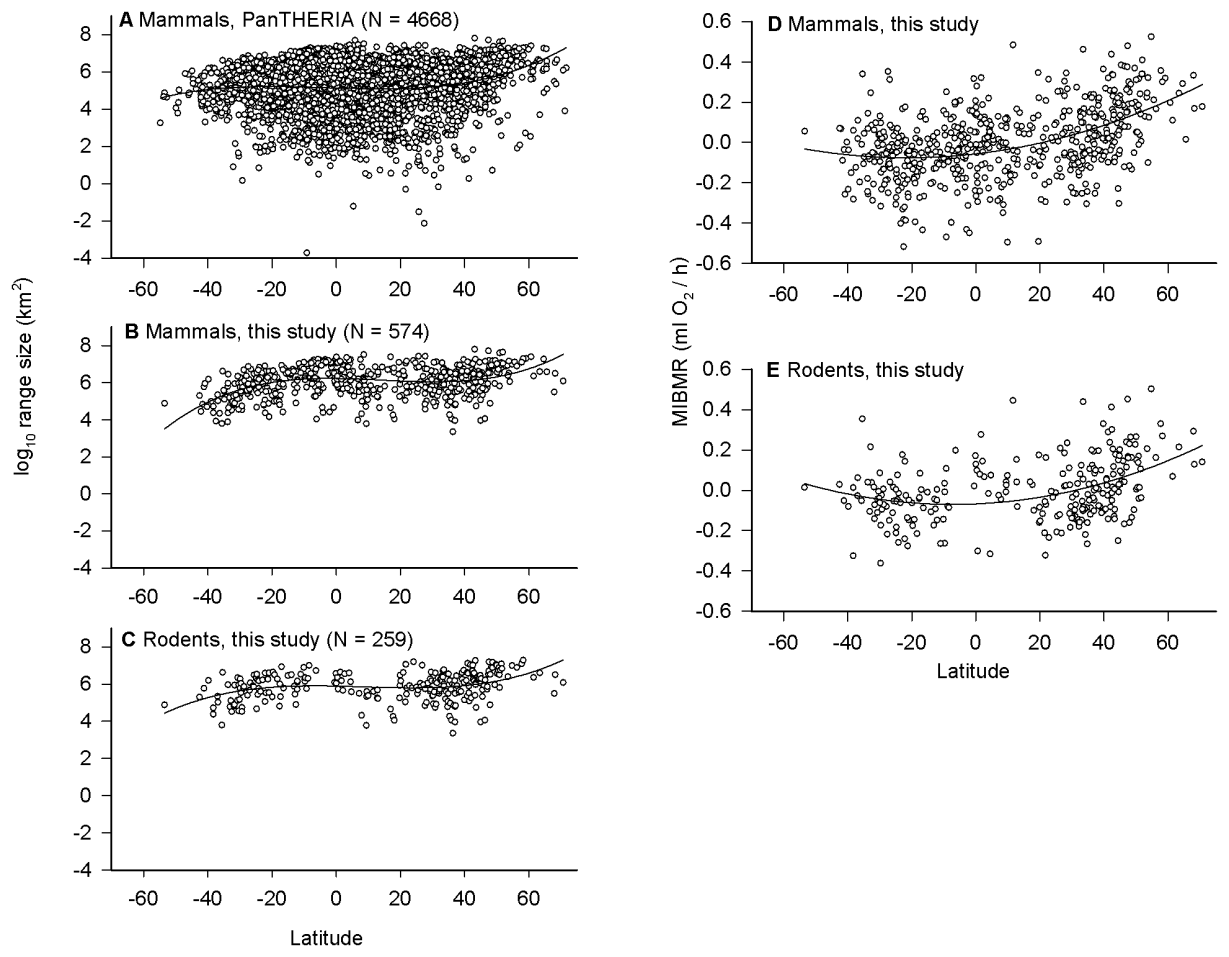
Latitudinal trends in (a-c) geographic range size and (d, e) mass-independent basal metabolic rate (MIBMR) for various mammal datasets. Points along the x-axis represent the latitudinal midpoint of a species’ geographic range. Significant relationships are indicated by OLS regression lines-of-best-fit. Positive latitudes represent the Northern hemisphere; negative latitudes represent the Southern hemisphere.

Based on AICc scores, the OLS relationship between range size and latitude (Figure 2a-c) was best described by a cubic function for all mammals (PanTHERIA: R^2^ = 0.025, F_3, 4664_ = 39.87, p < 0.0001), the subset of mammals in this study (R^2^ = 0.15, F_3, 570_ = 34.45, p < 0.0001), and the subset of rodents in this study (R^2^ = 0.11, F_3, 255_ = 10.78, p < 0.001). In each case, average range size increased in the Southern hemisphere from about -55° latitude towards the tropics, remained constant throughout the tropics, and increased from the tropics to high latitudes in the Northern hemisphere.

The relationship between MIBMR and latitude (Figure 2d,e) was best described by a quadratic function for all mammals (R^2^ = 0.20, F_2, 571_ = 73.20, p < 0.0001) and just rodents (R^2^ = 0.15, F_2, 256_ = 22.25, p < 0.0001). MIBMR increased relatively strongly with latitude in the Northern hemisphere, where the data extend to 70.7° latitude ( *Lemmus* *sibiricus* ). MIBMR increased less strongly (if at all) with latitude in the Southern hemisphere, where the data extend only to -53.5° latitude ( *Euneomys* *chinchilloides* ), with few data points past -40° latitude. Note that south of -55° latitude, there is essentially no land until reaching Antarctica.

### Mass-, BMR- and MIBMR-range size relationships

**Figure 3:**
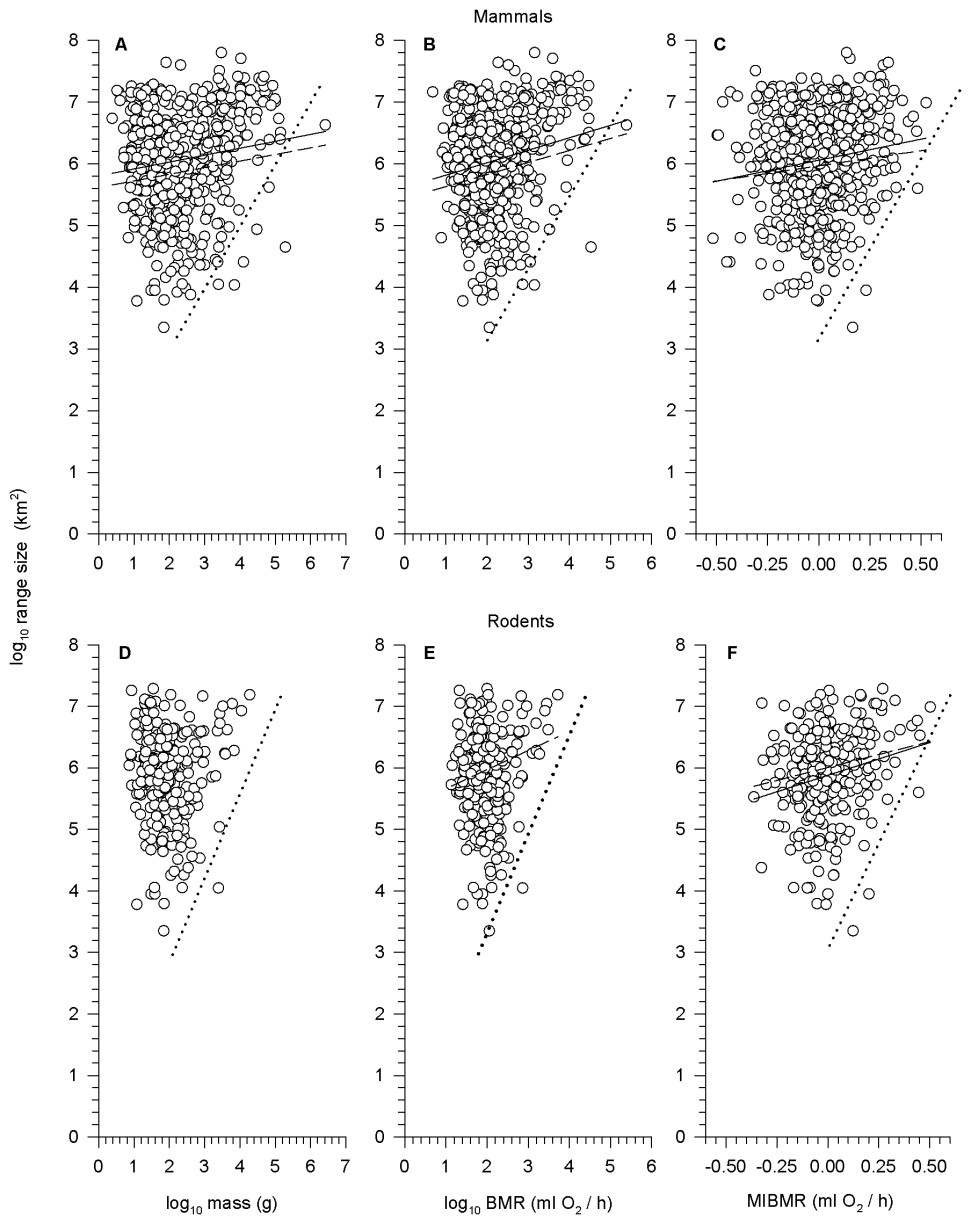
Among-species relationship between geographic range size and (a, d) mass, (b, e) BMR, and (c, f) MIBMR in all mammals and rodents. OLS (solid) and PGLS (dashed) regression lines are plotted where significant. Dotted lines were drawn by eye to illustrate the hypothesized functional constraint (see text).

**Figure 4:**
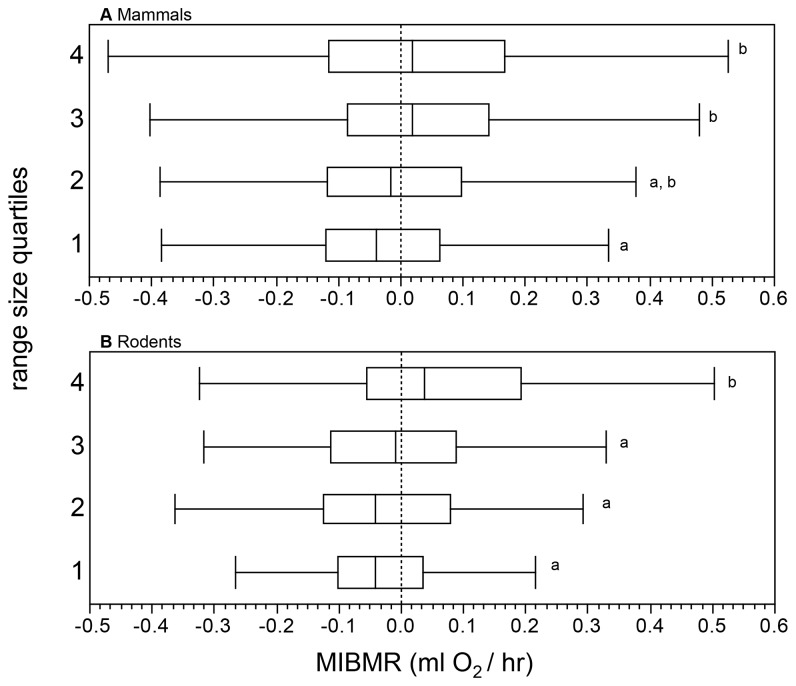
Box-plots of MIBMR among quartiles of geographic range size for (a) all mammals and (b) rodents. Different letters indicate significant differences among quartiles (ANOVA followed by Tukey-Kramer HSD test).

**Figure 5:**
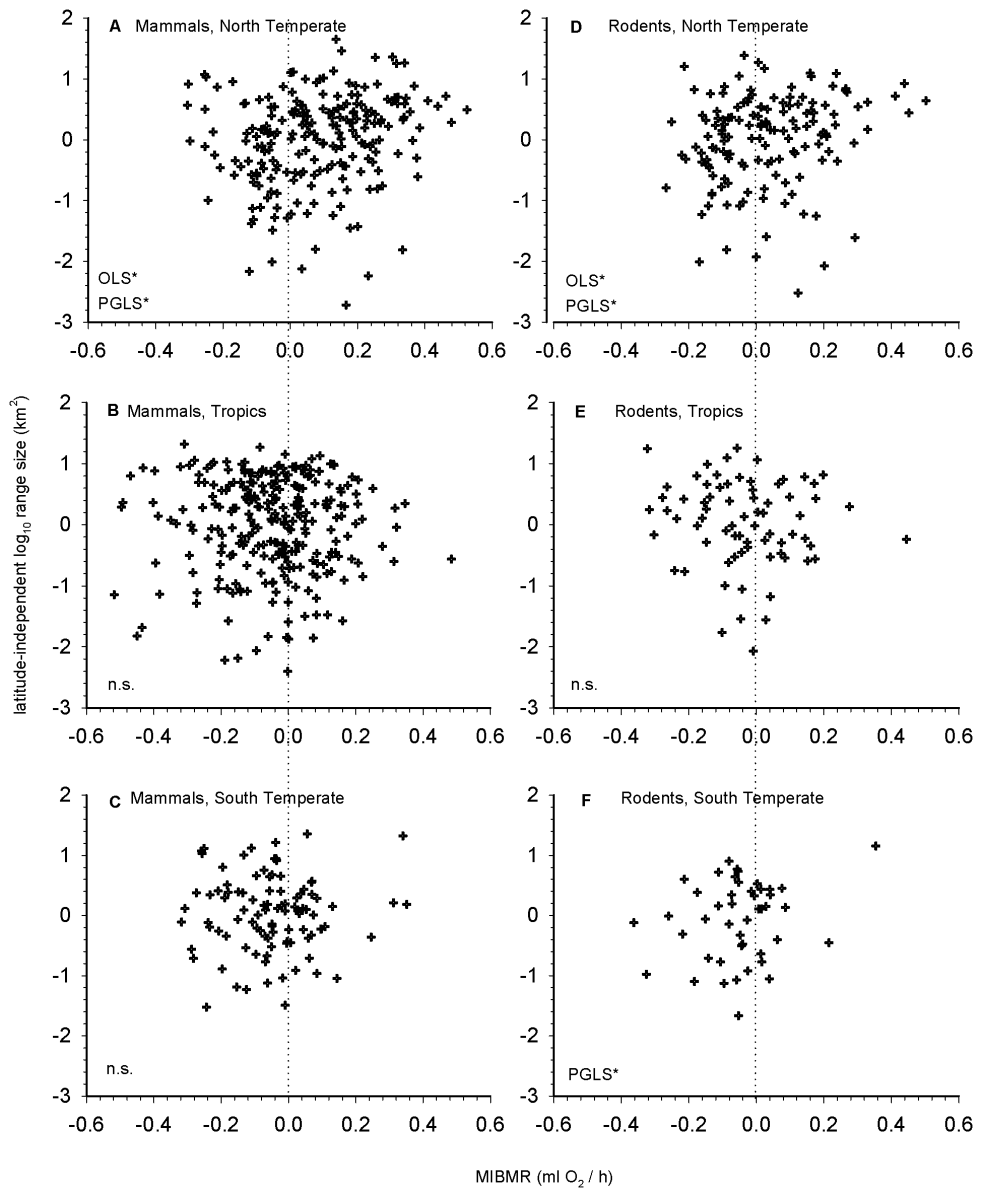
Among-species relationship between latitude-independent geographic range size and MIBMR for species whose range latitudinal midpoint occurs in the (a, d) North, (b, e) Tropics and (c, f) South for all mammals and rodents. Significant OLS and PGLS relationships are indicated by an asterisk.

For all mammals, there was a significant positive relationship between range size and mass, range size and BMR, and range size and MIBMR (Table 2; Figure 3a-c). Although the parameter estimates differed, the same results were found using OLS and PGLSλ regression (Table 2). We also divided the range size data into quartiles: average MIBMR decreased significantly from the highest to lowest quartile (Figure 4a). When the correlation between latitude and range size was removed, the relationship between latitude-independent range size and MIBMR was not significant for OLS and marginally significant (0.10 < p > 0.05) for PGLSλ (Table 2). However, when the data were analyzed separately by region (Table 3), there was a significant relationship between range size and MIBMR (Figure S3) and between latitude-independent range size and MIBMR (Figure 5a-c) in the North (n = 214) for both OLS and PGLSλ, whereas no relationship was detected in the Tropics (n = 263) or South (n = 97).

**Table 2:** Global non-phylogenetic (OLS) and phylogenetic (PGLSλ) simple linear regressions of geographic range size versus mass, BMR, and MIBMR in all mammals and rodents.

			Regression model:			
Group	Test	Parameters	OLS	p-value	PGLSλ	p-value
Mammals	GR~mass	slope	0.112 (0.033)	**<0.001**	0.107 (0.048)	**0.03**
		intercept	5.805 (0.082)	<0.001	5.620 (0.369)	<0.001
		*R* ^2^	0.02		-	
		*λ*	-		0.56	
		AICc	1385.99		1250.53	
	GR~BMR	slope	0.202 (0.045)	**<0.001**	0.195 (0.064)	**0.002**
		intercept	5.615 (0.105)	<0.001	5.438 (0.373)	<0.001
		*R* ^2^	0.03		-	
		*λ*	-		0.55	
		AICc	1377.95		1246.34	
	GR~MIBMR	slope	0.682 (0.194)	**<0.001**	0.491 (0.205)	**0.02**
		intercept	6.059 (0.034)	<0.001	5.971 (0.342)	<0.001
		*R* ^2^	0.02		-	
		*λ*	-		0.57	
		AICc	1385.32		1248.97	
	LIGR~MIBMR	slope	0.270 (0.180)	0.13	0.367 (0.196)	**0.06**
		intercept	-1.3e-11(0.031)	1.00	-0.019 (0.297)	0.95
		*R* ^2^	0.004		-	
		*λ*	-		0.49	
		AICc	1299.63		1206.81	
Rodents	GR~mass	slope	0.016 (0.076)	0.83	0.145 (0.099)	0.14
		intercept	5.848 (0.154)	<0.001	5.712 (0.277)	<0.001
		*R* ^2^	0.00		-	
		*λ*	-		0.38	
		AICc	597.90		586.91	
	GR~BMR	slope	0.145 (0.106)	0.17	0.310 (0.132)	**0.02**
		intercept	5.590 (0.216)	<0.001	5.353 (0.325)	<0.001
		*R* ^2^	0.01		-	
		*λ*	-		0.37	
		AICc	596.05		583.02	
	GR~MIBMR	slope	1.066 (0.304)	**<0.001**	0.842 (0.328)	**0.01**
		intercept	5.879 (0.046)	<0.001	6.007 (0.169)	<0.001
		*R* ^2^	0.05		-	
		*λ*	-		0.35	
		AICc	585.85		580.22	
	LIGR~MIBMR	slope	0.531 (0.291)	**0.069**	0.541 (0.314)	**0.086**
		intercept	-3.7e-11(0.004)	1.000	0.113 (0.147)	0.44
		*R* ^2^	0.013		-	
		*λ*	-		0.29	
		AICc	563.70		561.67	

All variables were log_10_ transformed. GR = geographic range size; BMR = basal metabolic rate; LIGR = latitude-independent GR; MIBMR = mass-independent BMR. Standard error of the slope (b) and intercept (a) are in parentheses. Statistically significant (p < 0.05) and “marginally significant” (0.10 < p > 0.05) slopes are indicated by p-values in bold.

**Table 3:** Regional non-phylogenetic (OLS) and phylogenetic (PGLSλ) simple linear regressions of geographic range size as a function of MIBMR in all mammals and rodents in the North (> 23.7° lat), Tropics (23.7° to -23.7° lat), and South (< -23.7° lat)

Response variable:			Range size				Latitude-independent range size			
			Regression model:				Regression model:			
Group	Region	Parameters	OLS	p-value	PGLSλ	p-value	OLS	p-value	PGLSλ	p-value
Mammals	North	slope	1.256 (0.305)	**<0.0001**	0.990 (0.322)	**0.0024**	0.802 (0.301)	**0.008**	0.655 (0.317)	**0.04**
		intercept	6.081 (0.058)	<0.0001	6.345 (0.304)	<0.0001	-0.068 (0.057)	0.23	0.215 (0.309)	0.48
		*R* ^2^	0.07		-		0.03		-	
		*λ*	-		0.38		-		0.38	
		AICc	492.33		465.48		486.46		456.20	
	Tropics	slope	0.065 (0.306)	0.83	0.039 (0.301)	0.90	-0.053 (0.298)	0.82	-0.020 (0.297)	0.95
		intercept	6.145 (0.052)	<0.0001	5.988 (0.428)	<0.0001	-0.011 (0.051)	0.85	-0.147 (0.407)	0.72
		*R* ^2^	0.0002		-		0.0001		-	
		*λ*	-		0.74		-		0.71	
		AICc	636.87		561.59		622.97		556.34	
	South	slope	-0.104 (0.547)	0.85	0.161 (0.537)	0.76	0.066 (0.484)	0.89	0.402 (0.461)	0.39
		intercept	5.549 (0.078)	<0.0001	5.629 (0.278)	<0.0001	0.029 (0.069)	0.68	0.089 (0.306)	0.77
		*R* ^2^	0.0004		-		0.0002		-	
		*λ*	-		0.37		-		0.55	
		AICc	214.99		215.58		191.21		187.44	
Rodents	North	slope	1.476 (0.396)	**<0.001**	1.249 (0.439)	**0.005**	0.908 (0.396)	**0.025**	0.899 (0.434)	**0.04**
		intercept	5.909 (0.066)	<0.0001	6.051 (0.162)	<0.0001	-0.023 (0.062)	0.71	0.134 (0.174)	0.44
		*R* ^2^	0.09		-		0.036		-	
		*λ*	-		0.23		-		0.28	
		AICc	320.98		321.47		318.39		315.23	
	Tropics	slope	-0.631 (0.589)	0.30	-0.029 (0.566)	0.95	-0.528 (0.572)	0.36	-0.280 (0.147)	**0.062**
		intercept	5.869 (0.085)	<0.0001	6.015 (0.267)	<0.0001	0.009 (0.086)	0.91	-0.776 (0.161)	<0.001
		*R* ^2^	0.015		-		0.012		-	
		*λ*	-		0.76		-		1.33	
		AICc	160.35		145.94		158.84		80.40	
	South	slope	0.734 (0.836)	0.39	0.874 (0.757)	0.25	1.132 (0.783)	0.16	1.432 (0.698)	**0.046**
		intercept	5.536 (0.107)	<0.0001	5.557 (0.232)	<0.0001	-0.010 (0.104)	0.92	-0.015 (0.219)	0.95
		*R* ^2^	0.02		-		0.046		-	
		*λ*	-		0.41		-		0.44	
		AICc	98.54		93.92		92.78		85.70	

All variables were log_10_ transformed. Standard error of the slope (b) and intercept (a) are in parentheses. Statistically significant (p < 0.05) and “marginally significant” (0.10 < p > 0.05) slopes are indicated by p-values in bold.

For rodents, there was no significant relationship between range size and mass or between range size and BMR (Table 2; Figure 3d,e). There was a significant positive relationship between range size and MIBMR for both OLS and PGLSλ regression (Table 2; Figure 3f). As with all mammals, average MIBMR decreased from the highest to lowest range size quartile (Figure 4b). When the effect of latitude on range size was removed, there was a marginally significant relationship between latitude-independent range size and MIBMR for both OLS and PGLSλ (Table 2). However, again, these relationships changed when separated by region (Table 3). As in the all-mammals dataset, there was a significant positive relationship between range size and MIBMR (Figure S3) and between latitude-independent range size and MIBMR (Figure 5d-f) for rodents in the North (n = 142). Unlike all mammals, and only for the PGLSλ analysis of latitude-independent range size, there also was a significant positive relationship in the South (n = 45).

The amount of variation in range size explained by MIBMR, mass and latitude combined varied from 2-28%, depending on geographic region and taxonomic scale (Table 4). For all mammals in the North, OLS multiple regression showed significant positive effects of each variable, while PGLSλ multiple regression revealed only a significant effect of latitude with marginally significant effects of mass and MIBMR. In the Tropics, only mass had a significant positive effect on range size in the OLS multiple regression; the PGLSλ multiple regression was not significant. In the South, only latitude had a significant negative effect on range size in both OLS and PGLSλ multiple regressions.

**Table 4:** Partial regression coefficients (b_*i*_) of non-phylogenetic (OLS) and phylogenetic (PGLSλ) multiple regressions of geographic range size as a function of MIBMR, mass and the range latitudinal midpoint in the North (> 23.7° lat), Tropics (23.7° to -23.7° lat), and South (< -23.7° lat)

Regression model:			OLS					PGLSλ				
Group	Region	Variable	b_i_	SE	p-value	Model R2	Model AICc	b_i_	SE	p-value	λ	Model AICc
Mammals	North	MIBMR	0.791	0.244	**0.02**	0.16	475.37	0.557	0.326	**0.09**	0.36	449.18
		mass	0.181	0.051	**0.001**			0.130	0.070	**0.06**		
		latitude	0.016	0.006	**0.01**			0.022	0.005	**<0.001**		
	Tropics	MIBMR	-0.01	0.311	0.97	0.02	636.33	0.032	0.303	0.91	0.72	562.46
		mass	0.094	0.046	**0.04**			0.068	0.066	0.30		
		latitude	0.003	0.004	0.47			-0.005	0.004	0.20		
	South	MIBMR	0.078	0.472	0.87	0.28	188.05	0.416	0.443	0.35	0.61	182.64
		mass	-0.022	0.071	0.75			0.085	0.084	0.31		
		latitude	-0.069	0.012	**<0.001**			-0.076	0.011	**<0.001**		
Rodents	North	MIBMR	1.051	0.443	**0.02**	0.13	332.26	0.894	0.448	**0.05**	0.28	323.24
		mass	0.023	0.107	0.83			0.153	0.128	0.23		
		latitude	0.018	0.008	**0.02**			0.024	0.008	**0.002**		
	Tropics	MIBMR	-0.521	0.589	0.38	0.08	172.68	-0.263	0.309	0.40	1.29	95.45
		mass	0.146	0.137	0.29			0.114	0.073	0.13		
		latitude	-0.008	0.006	0.12			-0.001	0.003	0.68		
	South	MIBMR	1.420	0.807	**0.09**	0.20	102.77	1.370	0.699	**0.06**	0.50	93.31
		mass	-0.133	0.153	0.39			0.312	0.188	0.11		
		latitude	-0.048	0.017	**0.01**			-0.052	0.016	**0.003**		

All variables were log_10_ transformed. Statistically significant (p < 0.05) and “marginally significant” (0.10 < p > 0.05) coefficients are indicated by p-values in bold.

For rodents in the North, both OLS and PGLSλ multiple regression showed significant positive effects of latitude and MIBMR on range size, but no effect of mass (Table 4). In the Tropics, there was no significant effect of any variable on range size. In the South, latitude had a significant negative effect and there was a marginally significant effect of MIBMR in both OLS and PGLSλ regression.

### Family-level analyses

**Figure 6:**
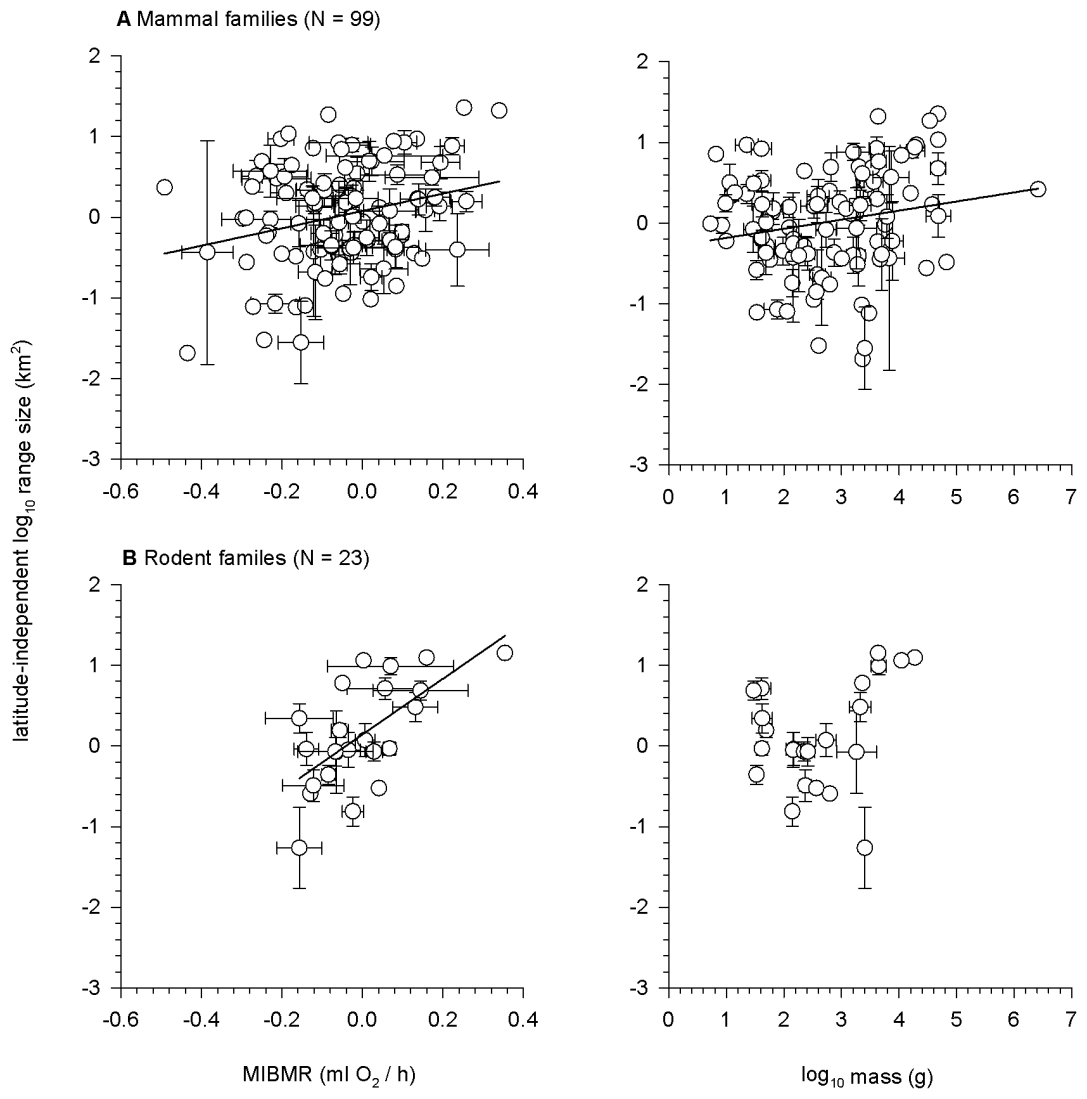
Among-families relationship between latitude-independent geographic range size and MIBMR and mass for (a) all mammal families and (b) rodent families only. Points are average values for a family ± 1 SE. Points without error bars are families represented by a single species. OLS regression lines are plotted where significant.

As described earlier, the low species-level resolution of mammalian relationships afforded weak tests of the impact of phylogeny upon the traits analyzed, so we opted to also examine these relationships at the family level where data permitted. For all mammal families (Figure 6a), there was a significant positive OLS relationship between mean MIBMR and mean latitude-independent range size (slope = 1.075 ± 0.402, R^2^ = 0.07, F_1, 97_ = 7.16, p = 0.009) and between mean mass and mean latitude-independent range size (slope = 0.112 ± 0.058, R^2^ = 0.04, F_1, 97_ = 3.84, p = 0.053). By contrast, for rodent families only (Figure 6b), there was a strong positive OLS relationship between mean MIBMR and mean latitude-independent range size (slope = 3.462 ± 0.896, R^2^ = 0.42, F_1, 21_ = 14.93, p = 0.0009), but no relationship between mean mass and mean latitude-independent range size (slope = 0.266 ± 0.156, R^2^ = 0.12, F_1, 21_ = 2.90, p = 0.103)

## Discussion

Geographic range size is codified as a key criterion for inferring vulnerability to extinction [107,108]. The underlying assumption is that narrow distribution or stenotopy is inherently risky and conversely that broad distribution or eurytopy confers resistance to stochastic extinction. Hence, the identification of species’ traits including PSTs that relate to range size is essential to assess vulnerability to extinction and, moreover, key to gaining a mechanistic understanding of range size diversity and dynamics [32,109].

### Metabolic rate, range size and the Energy Constraint Hypothesis

We hypothesized that diversity in geographic range size in the assemblage of contemporary terrestrial mammals would be positively related to diversity in metabolic rate. Using the largest macrophysiological dataset yet assembled for mammals (or any other major monophyletic radiation), we found evidence for this prediction in two dimensions: BMR, a measure of absolute minimal energy demand, and MIBMR, a measure of relative minimal energy demand, both of which are significantly positively correlated with range size.

Our analysis of the classic macroecological relationship between mass and range size showed that as body size increases, species occupy larger ranges, so there is a general lack of large-bodied stenotopic species (Figure 3a,d), as previously known [18,63,65,110]. Our new macrophysiological analyses showed a similar lack of stenotopic species with high BMRs (Figure 3b,e) or high MIBMRs (Figure 3c,f). In terms of our three hypothesized mechanisms (Figure 1), the observed patterns are most consistent with that expected from the Energy Constraint Hypothesis (Figure 1b) previously invoked to explain the relationship between mass and range size: small body size/low BMR or low MIBMR does not necessarily restrict species to smaller ranges, but large body size/high BMR or high MIBMR apparently constrains species to relatively large ranges. Presumably, the operant constraints on minimum range size in both dimensions of energetics stem from per capita energy demand, in which an increase in either absolute (mass, BMR) or relative (MIBMR) energy demand increases the minimum area required for individuals and therefore species to support minimum viable populations to avoid extinction.

A recent refinement of the Energy Constraint Hypothesis modifies the classic view of the body size-range size relationship in mammals [65]. That work, which examined a much larger body size-range size data set (n = 3268 species, approx. 57% of known species) than this or previous studies detected a transition in the body size-range size relationship around the modal mammal body size (~40 g in the expanded body size-range size data set [65]; ~100 g in the entire assemblage of mammals [111]). For larger species to the right of the mode, the pattern described above of progressively larger ranges with increasing body size was clearly evident in the expanded data set, consistent with the original Energy Constraint Hypothesis [18]. However, for smaller species to the left of the mode, the relationship between body size and range size was actually negative, prompting a modified Energy Constraint Hypothesis that includes a transition in the energetics of body size and its consequences for minimum space requirements as body size departs in either direction from the mode. Because the allometry of BMR to mass in mammals has a slope < 1, there is a transition from high mass-specific (i.e., per gram) BMR in small mammals to low mass-specific BMR in large mammals. Thus, the smallest mammals have the highest mass-specific energy demands, which may increase their space needs above those expected based solely on their small body size, as seen in the relationship between body size and home range size [112,113]. Thus, the negative relationship between body size and geographic range size in small mammals is now argued to be another constraint on minimum space requirements arising from progressively higher mass-specific energy demands at small body sizes [65]. In short, although the body size-range size relationships we present here fit the classic view of the triangular constraint space in Figure 1b, it is important to recognize that an expanded data set [65] shows the relationship actually shifts from negative in the smallest species (< ~ log_10_ [2] = 100 g) to positive in larger species (> log_10_ [2] =100 g).

#### Comparative explanatory power of mass and metabolic rate: analysis of central tendencies

It has long been assumed that energy demand underlies the relationship between mass and range size [18]. Our analysis of BMR and range size confirms this (Figure 3b,e). Moreover, our analysis of MIBMR uncovers a second, previously unrecognized dimension to the energetics of range size diversity in mammals. In fact, MIBMR explained more variation in range size than mass, the most widely studied macroecological predictor of range size [7,14,17,64,114,115] (Table 1). In OLS regressions, among-species variation in MIBMR explained from 2-5% of variation in range size, with and without the effects of latitude. Despite this small amount of explained variance, MIBMR explained almost twice the interspecific range variance than did mass (2% in mammals, none in rodents). This pattern was amplified in the family-level analyses. Among families, MIBMR explained 7% and 42% of the variation in latitude-independent range size in mammals and in rodents, respectively. By comparison, mass explained 4% of variation in latitude-independent range size among all mammal families and explained none of the range variance among rodent families. However, the small amount of variation explained by the predictors we examined highlights the limited success that traditional regression approaches have had in resolving determinants of variation in range size, especially when large numbers of species are compared within a single set of statistical models [14,61].

#### Comparison of boundary line-constrained trait spaces: analysis of functional diversity

A more informative perspective on macro-scale relationships between range size and species traits is the idea of a 2D trait-space bounded by mechanisms that restrict the combinations of values to fall within a constrained space (Figure 1). The ‘constraint space’ approach [14] shifts emphasis from estimating linear relationships between variables to considering why certain regions in any two-space are well-populated by extant species, whereas others have a dearth or even complete lack of species. Further, the constraint space approach aims to decipher the functional constraints that correlate with parts of the trait-space in which particular types of species are under-represented or non-existent.

From this perspective, we find that the shapes of the trait-spaces describing the relationships between range size and the predictor variables are highly similar (Figure 3). Each trait-space is roughly triangular and has a positive lower bound (dotted lines in Figure 3) indicating a constraint on minimum range size as a function of absolute (mass, BMR) and relative (MIBMR) energy demand. The finding that trait-spaces involving metabolic rates as predictors reveal the same positive lower bound previously observed for mass provides the first direct macrophysiological evidence that energy limitation per se constrains the lower right portion of the trait-space, supporting the Energy Constraint Hypothesis [14,18,62-65,115]. For BMR, this is not surprising because it is highly correlated with mass, which is what motivated the Energy Constraint Hypothesis to explain the body size-range size relationship in the first place [18]. Nevertheless, our analysis is apparently the first direct test using metabolic rate data.

The discovery of the triangular trait-space and positive lower bound in the MIBMR-range size dimension is unexpected and, because BMR and MIBMR are not highly correlated with each other, it reveals a second dimension of energetic constraint on mammal distributions. MIBMR can be thought of as the non-allometric interspecific variance in BMR. In the context of the Energy Constraint Hypothesis, the result that the positive lower bound is also evident for MIMBR indicates that species that have deviated towards higher metabolic rates are relatively rare and require among the largest ranges, above and beyond the increasing energy demands that accrue as an allometric consequence of evolutionary increases in body size. Figure 3c,f makes clear that low MIBMR does not necessarily restrict species to small ranges, but high MIBMR constrains species to large ranges, presumably to satisfy their sustained high per capita energy demands.

The hypothesis that energy demand per se restricts range size can be considered further by examining the distribution of range sizes for modal-sized mammals and how it varies as body size departs from the mode towards larger body size. First, consider two proxies of absolute energy demand, namely body size and non-mass-adjusted BMR. The modal body size in our data set occurs at log_10_(1.23) = 17 g for all mammals and log_10_(1.51) = 32 g for rodents. The modal body size in the entire mammal assemblage is ~100 g [111]. Based on the allometry of BMR to body size, the modal mammal body size can be construed as evolutionarily “favorable” or “optimal” in terms of the energetics of converting resources into reproductive power [116]. Inspection of Figure 3a,d shows that as body size evolves away from this modal size range towards larger body size, a constraint on the minimum area needed to support the species appears to arise almost immediately, a point made in seminal macroecologcial analyses of birds [18] and mammals [63]. Similarly, if viewed in terms of absolute energy demand (BMR; Figure 3b,e), the x-intercept of the lower constraint line appears to occur at ~log_10_ (2) = 100 mLO 2/h, which is the expected BMR for a ~log_10_ (2) = 100g mammal. Thus, an apparent constraint arises as mammal body size, and by allometric consequence, its absolute energy demand increases from the modal body size. Second, from the perspective of relative energy demand, we can ask how departures above and below expected BMR influence range size. Examination of the plots of range size versus MIBMR (Figure 3c,e; Figure S3) and latitude-independent range size versus MIBMR (Figure 5) suggest that the constraint line crosses the x-axis at ~0.0. Hence, as soon as MIBMR increases above zero, a constraint on minimum range size appears to arise such that mammals with elevated MIBMR (> 0.0) are constrained to have larger ranges.

### General insights from macro-scale analyses: geographic and phylogenetic scale

Relationships between species traits and measures of distribution and abundance, like those observed here, are generally weak in the traditional regression sense at large spatial and taxonomic scales for a variety of reasons [14,17,117]. Two findings from our study help explain some of this unresolved variance, namely, that there are hemispheric and temperate–tropic differences in functional relationships (Figure 2b, Figure 5, Table 4, Figure S3), and that the strength of functional relationships depends on phylogenetic scale (Figure 3, Figure 6).

#### Hemispheric differences in latitudinal patterns of range size

‘Rapoport’s rule’ describes an interspecific pattern in which range size increases with latitude [6,46]. Although this pattern has generally been found for terrestrial species in the Northern hemisphere, it is clearly not a rule because its existence is not taxonomically or geographically universal [7,27,118,119]. Where patterns consistent with Rapoport’s rule have been found, the most frequently proposed mechanism is the Climatic Variability Hypothesis [43].

Our analysis was the first to use all available data (4,668 species) from the PanTHERIA database [8] to examine the global latitudinal gradient in mammal range size and it revealed two notable patterns. First, based on the latitudinal midpoint method [7], we found that average range size increased with latitude in the Northern hemisphere from about 40° latitude towards the pole, but decreased in the Southern hemisphere from about -20° latitude towards the pole (Figure 2a-c). This hemispheric asymmetry affirms previous findings that Rapoport’s rule does not generalize across hemispheres [27,120].

Second, and more apparent than the trends in average range size, the variance in range size decreased with latitude in both hemispheres, so that there was a general absence of stenotopic species whose geographic ranges are centered at high latitudes in both hemispheres (Figure 2a). Thus, the global relationship between latitude and mammal range size can be summarized as: (1) few species with small ranges exist at high latitudes in both hemispheres, as predicted by the Climatic Variability Hypothesis (but also by the idea that the gradient in range size is linked to the gradient in species richness [46,120,121]), but (2) range size tends to decrease with latitude in the Southern hemisphere and increase with latitude in the Northern hemisphere, as predicted if available land area acts as a constraint. Indeed, land area decreases dramatically with latitude in the Southern hemisphere but increases with latitude in the Northern hemisphere [120].

A similar global pattern between range size and latitude occurs in birds, which was found to be well-correlated with the global latitudinal gradient in total land area (Figure 3 in [120]). Both land area and average range size increase with latitude in the Northern hemisphere where there is more land overall, but decrease with latitude in the Southern hemisphere where there is less land overall. In contrast, the Climatic Variability Hypothesis predicts a pattern of increasing range size with latitude in both hemispheres.

#### Hemispheric and tropic-temperate differences in functional relationships

Along with latitudinal variation in range size, multiple regression indicated that significant predictors of range size can be region-specific (Table 4). For all mammals and just rodents, the positive relationship between MIMBR and range size was most evident in the North where elevated MIBMR is most common (Figure 2d,e; see below). In this region, there was a significant positive relationship regardless of regression method (OLS vs. PGLS) or response variable (range size vs. latitude-independent range size) (Table 3). By contrast, the evidence for all mammals suggests no relationship between MIBMR and range size in the Tropics or South. For rodents, however, the relationship was significant in the South, but only for PGLS and not for OLS regression. An obvious question is: why might the positive MIBMR-range size relationship be most evident at high latitudes in the North?

The Climatic Variability Hypothesis predicts differences in physiological tolerances and, consequently, range sizes, between tropic versus temperate organisms [43]. MIBMR can be viewed as a proxy for thermal tolerance in endotherms because elevated metabolism increases the capacity for thermogenesis, which is needed to maintain body temperature at low environmental temperature (see section *Thermal Plasticity Hypothesis*). Consistent with this view and the predictions of the Climatic Variability Hypothesis, MIBMR increases with latitude, which explained 20% and 15% of the variation in all mammals and rodents, respectively (Figure 2c,e). These results corroborate a previous analysis of a smaller dataset (267 species) of small (< 1 kg) mammals [122]. Other aspects of metabolic capacity, such as nonshivering thermogenesis in rodents, also increase with latitude and decrease with environmental temperature [123]. However, while MIBMR clearly increases with latitude in the Northern hemisphere, it is not clear from our data whether this also occurs in the Southern hemisphere. Data are essentially absent in the Southern hemisphere past -50° latitude likely because the amount of land area decreases dramatically in this region [120] and species are unlikely to have geographic ranges centered at these latitudes. Past -55° latitude, there is essentially no land available until reaching the frozen Antarctic continent; the same latitude in the Northern hemisphere covers approximately 2 x 10^6^ km^2^ of land [120].

Figure 5 demonstrates more clearly differences in the distribution of MIBMR among the North, Tropics and South. Using MIBMR = 0.0 as a reference point, tropical species (Figure 5b,e) skew towards reduced MIBMR (n = 164, mean MIBMR = -0.143) versus elevated MIBMR (n = 99, mean = 0.106). By contrast, in the North (Figure 5a,d) more than twice as many species show elevated MIBMR (n = 147, mean = 0.174) as opposed to reduced MIBMR (n = 67, mean = -0.113). Thus, the comparison of tropical species to northern species provides strong empirical support for the Climatic Variability Hypothesis. However, the pattern does not hold for the South (Figure 5c,f), where twice as many species exhibit reduced MIBMR (n = 66, mean = -0.119) as opposed to elevated MIBMR (n = 31, mean = 0.093). This pattern is both opposite that of northern mammals and not consistent with the prediction of the Climatic Variability Hypothesis, a discrepancy that may relate to the different historical and contemporary patterns of climatic variability and cold temperatures experienced at high latitudes in the Northern (colder, more variable) versus Southern (warmer, less variable) hemispheres [27,28,76,119].

The fact that species with elevated MIBMR occur disproportionately in the North likely explains why we find clear evidence for a positive MIBMR-range size relationship in this region, but less evidence in the Tropics or South. In short, northern mammals tend to run “hot” with high relative energy demands, and thus they densely populate a region of trait-space close to the positive lower bound invoked by the Energy Constraint Hypothesis (Figure 1b).

#### Phylogeny

Most macroecological and macrophysiological analyses to date ignore the biases that arise from phylogenetic relatedness, which are widely acknowledged in many other areas of comparative biology [23,24]. In part this is due to the general lack of resolved phylogenies for the very large groups of species for which physiological or distributional data are assembled from the literature, but also by some concerns about the utility and interpretation of phylogenetic comparative methods.

Some have asserted that phylogenetic history should not be considered in the analysis of life historical or physiological traits because these traits must evolve rapidly and are under such strong adaptive constraint that it is not possible for phylogeny to constrain them [124-129]. However, these arguments have been mathematically falsified [130] or are inconsistent with other empirical findings of substantial lineage effects on BMR and its allometric scaling in both mammals and birds [60,82,131,132].

A more credible issue is that, although phylogenetic comparative methods aim to statistically separate the component of trait variation explained by common descent from that which is typically viewed as the adaptive component (i.e., that not correlated with phylogeny), phylogenetic signal is now known to also arise from strong adaptive stasis [100]. This finding means that because ‘phylogenetic signal’ actually includes a strong effect of adaptive evolution, current phylogenetic comparative methods over-compensate for phylogenetic inertia. Unfortunately, there is no current methodology for evaluating the contribution of adaptive stasis to phylogenetic signal, suggesting that phylogenetic comparative methods are overly conservative. Nonetheless, relatedness clearly imposes some inertial constraint on adaptive evolution that must be considered, even if current methods are overly conservative.

We employed the best-resolved (~50%) species level phylogeny in our analyses, but found that the results of non-phylogenetic and phylogenetic regression were qualitatively similar. However, AICc scores from phylogenetic regression were consistently lower or comparable to AICc scores from non-phylogenetic regression, indicating that accounting for phylogeny generally provided as good or a better fit to the data than not doing so. It must be noted that the overall poor resolution of the phylogeny means that the power of the phylogenetically-grounded analyses was weak. Thus, the lack of strong phylogenetic signal cannot be taken as a definitive indication that phylogeny does not play a role in character covariance among mammal species. This cautious view is reinforced by the additional analyses we conducted at the family level (Figure 6). While the overall phylogenetically-structured analyses were not strikingly different from those not accounting for phylogeny, the analysis at the family level revealed much stronger functional relationships than the species-level analysis (especially for rodents), which is a clear indication of covariance among species traits and range properties at the family level.

### The value of a macrophysiological approach: predictive power of PSTs and implications for climate change

Physiological Ecology is the field concerned with understanding how organisms transduce abiotic environmental variance into the phenotypes that determine both individual fitness and demographic dynamics [53-57,133-135]. Although it had a central role in early-mid 20th century Ecology, Physiological Ecology was seconded to Community Ecology (i.e., biotic interactions) as a principal focus of the study of species distributions [36]. Macrophysiology represents a conceptual reunification of physiology with ecology [36] and as such reintegrates these ideas into the ecological mainstream to address the fundamental ecological problem of understanding interspecific diversity in and functional constraints on the distribution and abundance of organisms. It also provides a theoretical platform for understanding mechanistically how abiotic factors influence distributions.

Our analysis of PSTs as predictors of mammalian distributions explained more variance than previous studies that have used more tangential predictors such as body size (Table 1), especially at the family level (Figure 6). Moreover, we found neglected but meaningful ecological signals in the residuals of the BMR data, indicating that relative energy demand, above and beyond absolute energy demand, explains additional variance in mammalian range sizes. This finding of latent signal in the residuals (MIBMR) reflects functional divergence (evolutionary excursions) of mammal species above and below the constraint imposed by allometric scaling, and shows that our current grasp of how physiological constraints relate to distributions in mammals and other lineages is incomplete. Neglected patterns in residuals of PST relationships deserve careful scrutiny in other systems and are fertile lines of inquiry for understanding species distributions [32]. Moreover, the macrophysiological signal emerging from the residuals invites caution in generalizations that have been advanced about how endotherms (or ectotherms) should respond to warming climates [136] and how range size relates to vulnerability to extinction.

The assumption underlying geographic range size as a key criterion for extinction vulnerability [107,108] is that small ranges (stenotopy) are inherently risky while large ranges (eurytopy) confer resistance to stochastic extinction, an assumption that may be sound, all else equal. However, our analyses show that the degree to which stenotopy is risky in mammals likely depends on body size and comparative energetics. The previously recognized absence of large-bodied, stenotopic species has been argued to be due to a relatively higher likelihood of lethal energy limitation due to the higher spatio-temporal unpredictability of energy in small geographic areas (Energy Constraint Hypothesis). Our analyses demonstrate directly the link between allometric and non-allometric increases in energy demand (estimated by BMR and MIBMR) and variation in range size. From this, we infer that the degree to which stenotopy is risky is greatest in large mammals with high absolute energy demand. Furthermore, we infer that because this pattern also holds in the dimension of relative energy demand, those mammals with higher than expected BMR for their body size are at heightened vulnerability to range size reductions, above and beyond the vulnerability accrued as a result of large body size or small range size. Hence, the mammal species most vulnerable to range size reductions and changes in energy landscapes (e.g., from habitat loss and climate change) are inferred to be those that are both large-bodied and have supra-allometric MIBMR, but also, small-bodied species with supra-allometric MIBMR are inferred to be at heightened risk. Other recent findings further suggest that the smallest mammals (those with the highest mass-specific BMR) are also at heightened risk from range size reductions [65]. Put another way, the mammal species least at risk from range size reductions will approximate the modal-sized mammal and have a low MIBMR.

Darwin’s [1] challenge to explain interspecific variance in range sizes among closely related species remains an active area of inquiry in modern Macroecology, made all the more pressing by the need to predict how species will respond to habitat loss and climate change. Macrophysiological approaches, which directly evaluate relationships between traits that are closely linked to organismal energetics and landscape-scale patterns of distribution, are useful for meeting this challenge, as shown here for analyses of the classic mammalian geographic range size distribution.

## Supporting Information

Figure S1 Histograms of geographic range size variation in (a-c) all mammals and (d-f) rodents. The dashed lines illustrate the bias in the data set used in this study (c,f) towards species with large ranges compared to the full distribution of range sizes found in the entire mammal assemblage (b, e).
(TIF)

Figure S2 Allometric relationship between mass and BMR in (a) all mammals and (b) rodents, including plots of the residuals (MIBMR) versus mass. (TIF)

Figure S3 Among-species relationship between geographic range size and MIBMR for species whose range latitudinal midpoint occurs in the (a,d) North region, (b,e) Tropics and (c,f) South region for all mammals and rodents. Significant OLS and PGLS relationships are indicated by an asterisk.
(TIF)
