# Supplementary figures and images for: A Macrophysiological Analysis of Energetic Constraints on Geographic Range Size in Mammals
Source: PLoS One. 2013 Sep 13;8(9):e72731. doi: 10.1371/journal.pone.0072731 (PMC3772909; doi:10.1371/journal.pone.0072731)

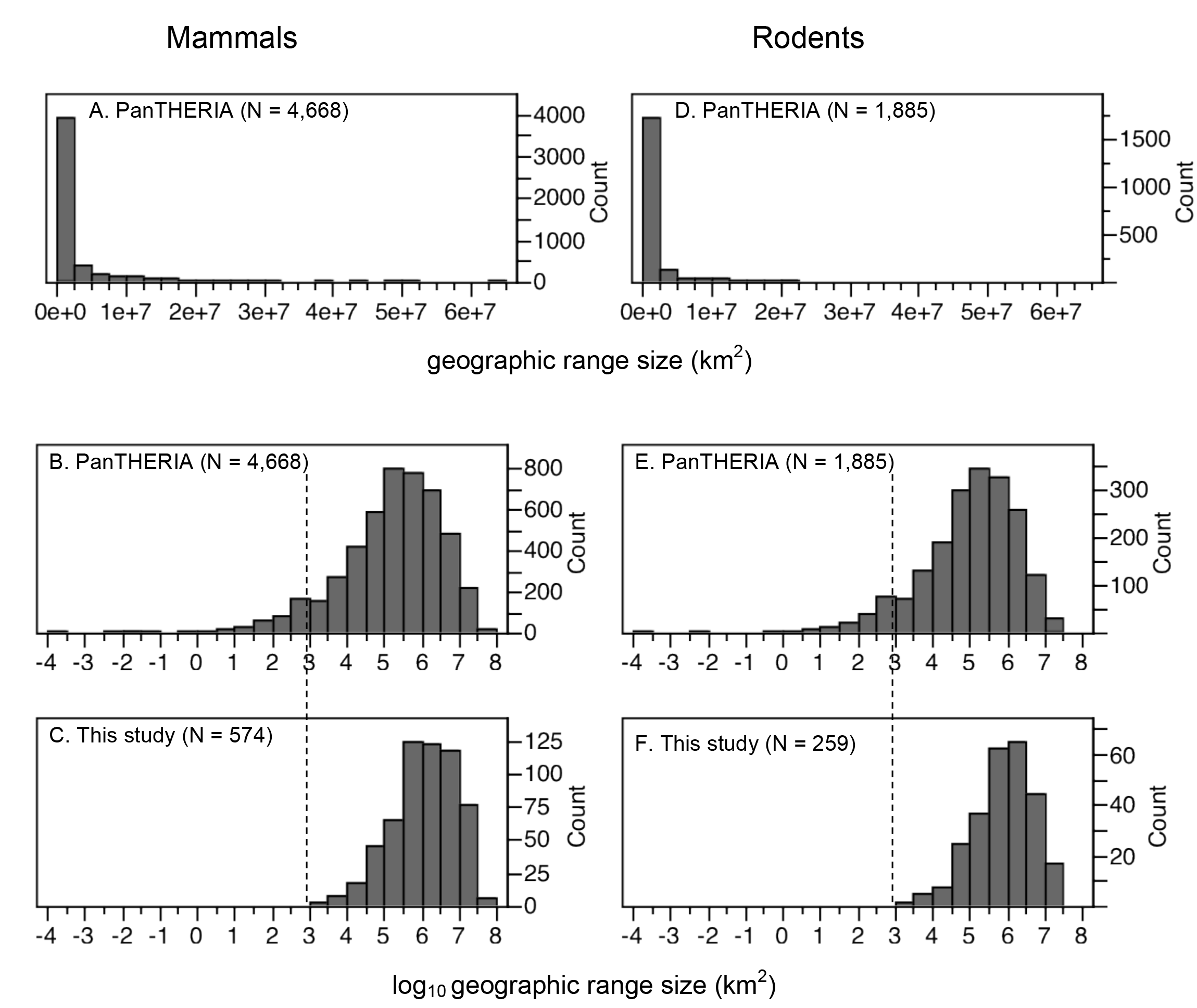

Supplement: Figure S1 — The dashed lines illustrate the bias in the data set used in this study (c,f) towards species with large ranges compared to the full distribution of range sizes found in the entire mammal assemblage (b, e). (TIF) [file pone.0072731.s001.tif]
